# Large-scale meta-analysis highlights the hypothalamic–pituitary–gonadal axis in the genetic regulation of menstrual cycle length

**DOI:** 10.1093/hmg/ddy317

**Published:** 2018-09-07

**Authors:** Triin Laisk, Viktorija Kukuškina, Duncan Palmer, Samantha Laber, Chia-Yen Chen, Teresa Ferreira, Nilufer Rahmioglu, Krina Zondervan, Christian Becker, Jordan W Smoller, Margaret Lippincott, Andres Salumets, Ingrid Granne, Stephanie Seminara, Benjamin Neale, Reedik Mägi, Cecilia M Lindgren

**Affiliations:** 1 Department of Obstetrics and Gynecology, Institute of Clinical Medicine, University of Tartu, Tartu, Estonia; 2 Estonian Genome Center, Institute of Genomics, University of Tartu, Tartu, Estonia; 3 Competence Centre on Health Technologies, Tartu, Estonia; 4 Analytic and Translational Genetics Unit, Department of Medicine, Massachusetts General Hospital and Harvard Medical School, Boston, Massachusetts, USA; 5 Stanley Center for Psychiatric Research, Broad Institute of Harvard and MIT, Cambridge, Massachusetts, USA; 6 Big Data Institute, Li Ka Shing Center for Health for Health Information and Discovery, Oxford University, Oxford, UK; 7 Wellcome Centre for Human Genetics, University of Oxford, Oxford, UK; 8 Psychiatric and Neurodevelopmental Genetics Unit, Massachusetts General Hospital, Boston, Massachusetts, USA; 9 Broad Institute of MIT and Harvard, Boston, Massachusetts, USA; 10 Oxford Endometriosis CaRe Centre, Nuffield Department of Women’s and Reproductive Health, University of Oxford, UK; 11 Harvard Reproductive Sciences Center and Reproductive Endocrine Unit, Massachusetts General Hospital, Boston, Massachusetts, USA; 12 Department of Obstetrics and Gynecology, University of Helsinki and Helsinki University Hospital, Helsinki, Finland; 13 Department of Biomedicine, Institute of Biomedicine and Translational Medicine, University of Tartu, Tartu, Estonia; 14 Nuffield Department of Women’s and Reproductive Health, University of Oxford, UK; 15 Program in Medical and Population Genetics, Broad Institute, Boston, MA, USA

## Abstract

The normal menstrual cycle requires a delicate interplay between the hypothalamus, pituitary and ovary. Therefore, its length is an important indicator of female reproductive health. Menstrual cycle length has been shown to be partially controlled by genetic factors, especially in the follicle-stimulating hormone beta-subunit (*FSHB*) locus. A genome-wide association study meta-analysis of menstrual cycle length in 44 871 women of European ancestry confirmed the previously observed association with the *FSHB* locus and identified four additional novel signals in, or near, the *GNRH1*, *PGR*, *NR5A2* and *INS-IGF2* genes. These findings not only confirm the role of the hypothalamic–pituitary–gonadal axis in the genetic regulation of menstrual cycle length but also highlight potential novel local regulatory mechanisms, such as those mediated by *IGF2*.

## Introduction

A menstrual cycle is crucial for human reproduction as it is required for oocyte selection, maturation and ovulation in preparation for its fertilization and subsequent pregnancy (1). The median menstrual cycle length is 27–30 days, depending on age (2) and can be divided into two distinct ovarian phases—the follicular and luteal phases separated by ovulation. During the follicular phase the emerging follicle secretes estrogen that causes proliferation of the endometrium, the uterine lining, and in the subsequent luteal phase progesterone secretion from the corpus luteum of the ruptured follicle causes endometrium to cease proliferating and change both phenotypically and functionally in preparation for implantation of the embryo (3). The menstrual cycle and its length are under the control of reproductive hormones secreted via the integration of the hypothalamic–pituitary–gonadal axis (HPG axis), where the gonadotropin-releasing hormone (GnRH) secreted from the hypothalamus stimulates the release of the gonadotropins, follicle-stimulating hormone (FSH) and luteinizing hormone (LH), from the anterior pituitary (3,4). FSH and LH in turn stimulate follicular growth and secretion of estrogens to prepare for ovulation and progesterone from ovarian follicular cells (3,4). The length of menstrual cycle reflects fertility status and has been associated with a range of reproductive traits, such as time to pregnancy, risk of spontaneous abortion and success rates in assisted reproduction (5–7). Moreover, shorter cycles have been associated with an increased risk of a gynecological condition known as endometriosis (8). Although a small twin study suggested no significant heritability for menstrual cycle length (9), it was recently demonstrated that a genetic variant in the promoter of follicle-stimulating hormone beta subunit gene (*FSHB*) is associated with longer menstrual cycles, nulliparity and lower endometriosis risk (10). However, only variants in, or near, the *FSHB* gene reached genome-wide significance among 9534 women (10), leaving the possibility that additional loci regulating menstrual cycle length could be revealed in larger studies.

Here, we present the results of a genome-wide association study (GWAS) meta-analysis of 44 871 women of European ancestry. We confirm the previous association with the *FSHB* locus (10) and also identify four additional novel association signals, contributing to an increase in our knowledge on the underlying genetics of menstrual cycle length control along the hypothalamus–pituitary–ovarian axis and also providing a genetic basis for the observed epidemiological correlations with gynecological pathologies.

## Results

### Genome-wide association signals for menstrual cycle length

**Figure 1:**
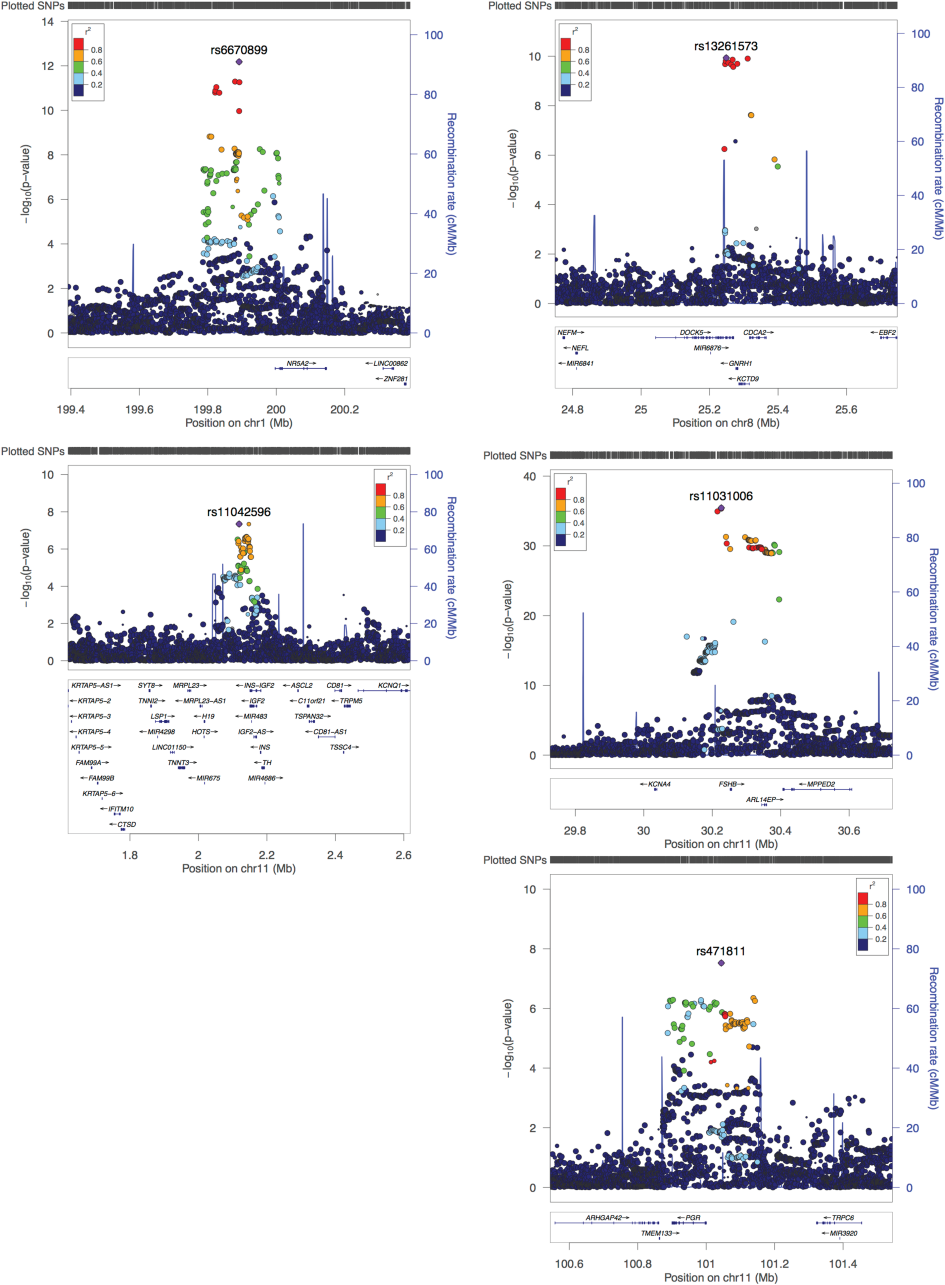
Regional plots for five genome-wide significant loci. Regional plot depicts SNPs plotted by their position and GWAS meta-analysis –log10 (*P*-value) for association with menstrual cycle length. Nearby genes are shown on the lower panel.

A total of five loci reached genome-wide significance (linear regression, *P* < 5 $\times$ 10^-8^) for association with menstrual cycle length in the meta-analysis, including data from two cohorts and a total of 44 871 women (Table 1, Fig. 1 and Supplementary Material, Fig. 1). The strongest signal [rs11031006, *P*_meta_ = 3.6 $\times$ 10^-36^, $\beta$_UKBB_ = −0.16 (s.e. = 0.01)] is in strong LD (*r*^2^ = 0.80) with the previously reported variant in *FSHB* promoter (rs10835638), while the remaining four loci are signals previously not reported. The strongest novel association [rs6670899, *P*_meta_ = 6.6 $\times$ 10^-13^, $\beta$_UKBB_ = −0.06 (s.e. = 0.01)] is 105 kb upstream of the *NR5A2* gene, which encodes a DNA-binding zinc finger transcription factor that is implicated in regulation of steroidogenesis during granulosa cell differentiation (11). This same region has previously been associated with age at menarche (12) [lead signal rs6427782 A-allele (*r*^2^ = 0.45 with rs6670899) was shown to increase age at menarche (12) and increases menstrual cycle length in our analysis, *P*_meta_ = 4.7 $\times$ 10^-6^]. The second novel signal [rs13261573, *P*_meta_ = 1.2 $\times$ 10^-10^, $\beta$_UKBB_ = −0.07 (s.e. = 0.01)] is in the second intron of the *DOCK5* gene, but in strong LD (*r*^2^ = 0.90) with rs6185 (*P*_meta_ = 2.0 $\times$ 10^-10^), a missense variant in the gonadotropin-releasing hormone 1 gene (*GNRH1*). *GNRH1* encodes the precursor for a peptide in the gonadotropin-releasing hormone family that regulates the release of FSH and LH from the anterior pituitary (3,4). We also observed two additional signals on chromosome 11; the first [lead signal rs471811, *P*_meta_ = 3.0 $\times$ 10^-8^, $\beta$_UKBB_ = −0.03 (s.e. = 0.01)] lies 42 kb upstream of progesterone receptor gene (*PGR*) and 14 kb downstream of a PGR antisense RNA (*PGR-AS1*). The second novel signal on chromosome 11 [rs11042596, *P*_meta_ = 4.5 $\times$ 10^-8^, $\beta$_UKBB_ = 0.04 (s.e. = 0.01)], is located 31 kb downstream the *INS-IGF2* and *IGF2* genes.

**Table 1:** Genetic variants associated with menstrual cycle length

Region	Nearest gene(s)	SNP	Alleles, other allele/effect allele (EAF)	UKBB		EGCUT		Meta-analysis	
				Effect (SD of the binned menstrual cycle length)	P-value	Effect (SD of the binned menstrual cycle length)	*P*	*P*	*P* heterogeneity
11:30226528	*FSHB*	rs11031006	A/G (0.86)	−0.16 (0.01)	1.1 × 10^−38^	−0.06 (0.02)	6.6 $\times$ 10^−4^	3.6 $\times$ 10^−36^	3.7 × 10^−6^
1:199891438	*NR5A2*	rs6670899	A/C (0.57)	−0.05 (0.01)	1.1 × 10^−10^	−0.04 (0.01)	4.7 $\times$ 10^−4^	6.6 $\times$ 10^−13^	0.43
8:25248615	*DOCK5/* *GNRH1*	rs13261573	A/G (0.75)	−0.07 (0.01)	1.7 × 10^−11^	−0.02 (0.01)	7.0 $\times$ 10^−2^	1.2 $\times$ 10^−10^	0.02
11:101044203	*PGR*/*PGR-AS1*	rs471811	C/T (0.31)	−0.03 (0.01)	4.8 × 10^−5^	−0.06 (0.01)	6.3 $\times$ 10^−5^	3.0 $\times$ 10^−8^	0.33
11:2118860	*IGF2/INS-IGF2*	rs11042596	G/T (0.34)	0.04 (0.01)	1.1 × 10^−7^	0.02 (0.01)	3.5 $\times$ 10^−2^	4.5 $\times$ 10^−8^	0.21

SD - standard deviation.

### SNP-based heritability of menstrual cycle length

We evaluated single nucleotide polymorphism (SNP)-based heritability (phenotypic variance explained by SNPs in the GWAS meta-analysis) using LD-score regression (LDSC) (13). The overall SNP-based heritability of menstrual cycle length was estimated at 6.1% (s.e. = 1.2). After filtering out all variants within $\pm$ 500 kb of the lead SNPs, the heritability estimate for menstrual cycle length decreased to 5.4% (s.e. = 1.1), indicating that common SNPs explain a small but significant part of menstrual cycle length variability, and moreover, the majority of the SNP-heritability still remains to be discovered.

### Gene-based associations of menstrual cycle length

A Multi-marker Analysis of GenoMic Annotation (MAGMA) (14) genome-wide gene association analysis of our GWAS meta-analysis summary statistics highlighted 10 genes that passed the suggested threshold for significance (*P* = 2.7 $\times$ 10^-6^, Bonferroni correction for association testing of 18 297 protein coding genes): *ARL14EP*, *SMAD3*, *MPPED2*, *RHBDD1*, *IGF2*, *COL4A4*, *PGR*, *INS-IGF2*, *FSHB* and *ARHGEF3* (Supplementary Material, Table 1). Six of these genes (*ARL14EP/FSHB/MPPED2, IGF2, INS-IGF2* and *PGR*) overlap with three loci identified in the single-marker analysis, while the remaining four novel gene signals did not harbor genome-wide significant SNPs (lowest *P*-values for SNPs in *SMAD3, RHBDD1, COL4A4* and *ARHGEF3* were rs11856909, *P* = 6.2 $\times$ 10^-8^*;* rs4673173, *P* = 1.0 $\times$ 10^-7^; rs12467261, *P* = 1.3 $\times$ 10^-7^; and rs73086331, *P* = 1.9 $\times$ 10^-6^, respectively).

### Genetic associations between menstrual cycle length and other traits

To evaluate the potential shared genetic architecture between menstrual cycle length and other traits, we performed a look-up in the GWAS catalogue (https://www.ebi.ac.uk/gwas/; Supplementary Material, Table 2) for menstrual cycle length associated variants and candidate SNPs identified by the Functional Mapping and Annotation of Genome-Wide Association Studies (FUMA) tool. Several significant associations were found for the *FSHB* locus, including gonadotropin (FSH and LH) levels, age at menarche and menopause, spontaneous dizygotic twinning, endometriosis and polycystic ovary syndrome (PCOS) (*P* <= 3 $\times$ 10^-8^). Additionally, the *NR5A2* locus was associated with menarche timing (*P* = 5 $\times$ 10^-8^) and showed some evidence for association with age at voice drop (*P* = 6 $\times$ 10^-7^) and pancreatic cancer (*P* = 1 $\times$ 10^-11^).

Next, to determine whether other phenotypes were associated with loci regulating menstrual cycle length, we conducted a PheWAS using the sentinel markers for each locus (rs11031006, rs6670899, rs13261573, rs471811 and rs11042596) and the UK Biobank (UKBB) phenotypes present in the Oxford Brain Imaging Genetics (BIG) browser (http://big.stats.ox.ac.uk/). Associations with a *P* < 2.1 $\times$ 10^-5^ (corresponding to a Bonferroni-corrected threshold of 0.05/2419) are shown in Supplementary Material, Table 3. Again, the *FSHB* locus (rs11031006) showed the largest number of associations, including three genome-wide significant associations (*P* < 5 $\times$ 10^-8^) with ‘Years since last cervical smear’, ‘Bilateral oophorectomy (both ovaries removed)’, ‘Diagnoses - main ICD10: N92 Excessive, frequent and irregular menstruation’ in UKBB. Nominally significant associations were also observed for ‘Age when periods started (menarche)’ (*P* = 1.7 $\times$ 10^-7^), ‘Non-cancer illness code, self-reported: endometriosis’ (*P* = 3.8 $\times$ 10^-7^), ‘Part of a multiple birth’ (*P* = 4.6 $\times$ 10^-7^), supporting the findings from the GWAS catalogue look-up. In this comparison, the allele associated with longer cycles decreased the risk of oophorectomy, menstrual cycle disturbances and endometriosis and was associated with later menarche. Similarly, rs6670899 (*NR5A2*) menstrual cycle-lengthening allele was associated with later menarche timing (*P* = 3.2 $\times$ 10^-8^).

Since the menstrual cycle and its disturbances are an important part of PCOS symptoms, we additionally performed a look-up of the reported PCOS susceptibility loci (15–19) and observed nominally significant (*P* < 0.05) associations with five loci (*FSHB*, *FSHR*, *RAB5B/SUOX*, *IRF1/RAD50* and *KRR1*) (Supplementary Material, Table 4).

Finally, we carried out a genetic correlation analysis with the LDSC method implemented in LD-Hub (20). Comparison with cardiometabolic, anthropometric, autoimmune, hormone, cancer and reproductive traits [for example lowest *P*-values were observed for age of first birth (r_g_ = 0.12, s.e. = 0.07, *P* = 0.055) and age at menopause (r_g_ = 0.15, s.e. = 0.08, *P* = 0.058)] revealed no significant correlations (Supplementary Material, Table 5).

### Functional annotation of associated variants and candidate gene mapping

Functional mapping and annotation of genetic associations for menstrual cycle length was carried out using FUMA (21), and a total of 600 candidate SNPs (defined as being in LD with the lead SNPs with a *r*^2^ >= 0.6) were identified. The majority of these (∼90%; Supplementary Material, Fig. 2A and Table 6) were located in intergenic or intronic regions, and >75% of the variants overlapped chromatin state annotations (Supplementary Material, Fig. 2C and Table 6), suggesting that they affect gene regulation.

To identify the potential effector transcripts for the five significant loci for menstrual cycle length, genes within the loci were prioritized if there was evidence for both expression quantitative trait loci (eQTL) and chromatin interaction (21).

In the *FSHB* locus, a total of 2 lead SNPs (rs11031006 and rs11032051), 8 independent (*r*^2^ < 0.6) significant SNPs and 359 candidate SNPs were identified (Supplementary Material, Table 6). Numerous significant eQTL associations (FDR < 0.05) were identified in different data sets (Supplementary Material, Table 7), but genes that were highlighted by both eQTL and chromatin interaction mapping included *FSHB*, *ARL14EP* and *MPPED2* (Supplementary Material, Fig. 3)*.*

The *INS-IGF2* locus (lead signal rs11042596) included a total of 34 candidate SNPs, with the lowest Regulome DataBase (RDB) score (1d—likely to affect binding and linked to expression of a gene target) for rs6578986. eQTL mapping and chromatin interactions highlighted *IGF2* and *INS-IGF2* as likely effector transcripts at this locus (Supplementary Material, Table 7 and Fig. 3).

The *PGR* locus (lead signal rs471811) included a total of 61 candidate SNPs, and *ANGPTL5* was prioritized by both eQTL [thyroid in GTEx_v7 (22); FDR < 0.05] and chromatin interaction analysis.

In the *NR5A2* locus on chromosome 1 (lead variant rs6670899), 4 independent significant SNPs and 133 candidate SNPs were identified (Supplementary Material, Table 6), 6 of which have evidence for likely affecting regulatory element binding (RDB score 2; Supplementary Material, Table 6). Two genes were prioritized based on eQTL data [*ZNF281* in dorsolateral prefrontal cortex (23) and C1orf106 in testis (GTEx_v7 (22); FDR < 0.05), while *ZNF281* was also additionally mapped using chromatin interaction data].

Finally, in the *DOCK5-GNRH1* locus on chromosome 8 (lead variant rs132661573), 13 potential candidate SNPs were identified (Supplementary Material, Table 6), including rs6185, a missense variant in the *GNRH1* gene. Seven of the 13 candidates are also eQTLs for *GNRH1* in whole blood (GTEx_v7 (22), FDR < 0.05).

### Tissue specificity and gene set enrichment analysis

Using the list of genes that were highlighted either in gene-based analysis and/or had both eQTL and chromatin interaction data supporting their candidacy, we performed a tissue specificity and pathway enrichment analysis with the GENE2FUNC option implemented in FUMA (21). Enrichment test of differentially expressed genes (DEGs) across GTEx_v7 30 tissue types (see Materials and methods) showed significantly higher expression of prioritized genes in female reproductive tissues: uterus (Bonferroni corrected *P*-value; *P*_Bon_ = 0.047), cervix uteri (*P*_Bon_ = 0.048) and ovary (*P*_Bon_ = 0.050; Supplementary Material, Fig. 4 and Table 8). Prioritized genes were also overrepresented in hormone activity-related pathways [for example, GO hormone activity FDR = 7.6 $\times$ 10^-7^, KEGG GnRH signaling pathway FDR = 1.5 $\times$ 10^-3^, WikiPathways (24) ovarian infertility genes FDR = 7.5 $\times$ 10^-5^ (Supplementary Material, Table 9)]. Tissue and cell-type enrichment analysis with DEPICT (25) revealed no significant enrichments.

Using GREAT (26) we found that genes within the five significant menstrual cycle length GWAS loci are enriched for uterus and circulating hormone level-related mouse phenotypes (Supplementary Material, Table 10) and further highlighted an enrichment at these loci for ‘genes involved in hormone ligand-binding receptors’ (*P*_FDR_ = 1.3 $\times$ 10^-2^; Supplementary Material, Table 11). Reviewing the MGI mouse phenotype database (27) showed that mouse knockouts of *Fshb, Nr5a2, Gnrh1* and *Pgr* all present with female reproductive phenotypes (Supplementary Material, Table 12), including altered estrous cycle length or abnormal ovulation for *Fshb, Gnrh1* and *Pgr* (progesterone receptor) (Supplementary Material, Table 13)*. Nr5a2* (nuclear receptor subfamily 5, group A, member 2) is linked to reduced fertility, primarily by reduced circulating progesterone levels in *Nr5a*2+/- female mice (28).

The presence of female reproductive phenotypes in mice with altered expression of *Fshb, Nr5a2, Gnrh1* and *Pgr* provides evidence that these genes may be causal and could explain, at least in part, the mediating mechanisms underlying four of the five significant loci associated with menstrual cycle length. Further experimental validation will be necessary to fully unravel the mechanism of these non-coding associations.

## Discussion

This large-scale GWAS meta-analysis reveals several novel insights into the genetic control of menstrual cycle length and provides evidence of the genetic underpinnings of the epidemiological associations between menstrual cycle length and other traits. Understanding the genetics regulating normal menstrual cycle variation is vital for figuring out the mechanisms leading to different menstrual cycle-related pathologies. Moreover, genetic control of menstrual cycle and folliculogenesis is of importance for *in vitro* fertilization treatment, where markers allowing for individualization of treatment protocols are still extensively sought (29).

While some of the results confirm what is already known about the biology of the menstrual cycle (such as the regulatory role of GnRH and FSH in the HPG axis), others point to potentially novel modulators and the role of local control of folliculogenesis. For example, IGF2 has been proposed to be an important local regulator of folliculogenesis (30) as it stimulates estrogen production (31) and modulates the action of FSH and LH, whereas IGF2 expression in turn is regulated by FSH (32). However, to our knowledge no direct link between genetic variation in the *INS-IGF2* region and menstrual cycle length had been previously demonstrated. Similarly, while it is known that progesterone is the dominant hormone in the second half of the menstrual cycle, the evidence linking genetic variation in the progesterone signaling pathway with menstrual cycle length was scarce (33,34). SMAD3, highlighted in gene-based analysis, is shown to modulate the proliferation of follicular granulosa cells and also ovarian steroidogenesis (35) and is an essential regulator of FSH signaling in the mouse (36). Recently, genetic variation in *SMAD3* was associated with dizygotic twinning (37). However, the obvious candidacy and support for one gene in most of these loci does not exclude the possibility that there might be additional genes and/or functional sequence in these loci that contribute to menstrual cycle length.

Analysis of pleiotropy between menstrual cycle length-associated variants and GWAS signals of other traits confirmed the central role of the *FSHB* locus, which is involved in regulating the reproductive lifespan from menarche to menopause and is also associated with gynecological diseases such as PCOS and endometriosis and with menstrual cycle disturbances. Additionally, we found nominally significant associations with some of the reported PCOS susceptibility loci (15–19), which might help understand how these loci are involved in PCOS pathogenesis. However, it should be emphasized that women with self-reported irregular menstrual cycles (a hallmark characteristic of PCOS) were excluded from the analyses, potentially limiting the overlap.

While there is epidemiological evidence that shorter menstrual cycles are associated with earlier age at menopause (38), we did not observe a significant overlap on a genetic level, as these traits did not show a significant genetic correlation. At the same time, the *FSHB* locus is significantly associated with both menstrual cycle length and age at menopause (10,39), indicating that this locus is probably largely driving the observed phenotypic correlation between menstrual cycle length and age at menopause in the literature.

Our study has a number of limitations. First, only self-reported data were available for menstrual cycle length, which might be difficult to accurately recall. Second, the UKBB includes women >40 years, some of whom are approaching menopause and might therefore have more irregular and shorter cycles (40), characteristic to the perimenopause. Therefore, a certain effect of the perimenopause on the effect sizes observed in UKBB cannot be ruled out, especially for the *FSHB* locus, where we observed significant heterogeneity in the effect estimates for the two cohorts. Also, participants in the UKBB were asked about their *current* cycle length, whereas EGCUT participants were asked to report their cycle length at the age of 25–35 years, where it is believed to be most regular (40). Although it is possible that the effect estimates from these two cohorts may not be directly comparable, we observe consistency in effect direction and magnitude. Third, we cannot rule out the possibility that some women in our sample have reported their cycle length during use of hormonal contraceptives or others hormones, which affect menstrual cycle length. Finally, while our sample size is the largest to date, it may still be underpowered to detect further associations.

In conclusion, the largest menstrual cycle length GWAS meta-analysis to date not only confirms the role of key players in the HPG axis in the genetic regulation of menstrual cycle length (*GNRH1*, *FSHB* and *PGR*) but also pinpoints novel genes with a potential local regulatory role (such as *IGF2*/*INS-IGF2* and *NR5A2*). Our analysis also highlights the central role of the *FSHB* locus in female reproductive health and provides evidence that the systemic determinants of normal menstrual cycle length (*FSHB*) are also associated with menstrual cycle-related pathologies, such as excessive, frequent and irregular menstruation. However, the loci identified as significant in our analysis represent a small fraction of the SNP-heritability for menstrual cycle length, warranting additional larger meta-analysis efforts to further uncover the remaining genetic underpinnings of menstrual cycle length. Additionally, we believe the current exploratory analysis forms a good basis for further similar studies with more refined research questions, such as the role of the identified variants in regulating cycle length at different stages of a woman’s life.

### Data availability

Summary statistics of single-marker analyses are available at http://www.geenivaramu.ee/tools/Cycle_length_Laisk_et_al_2018.gz.

## Materials and methods

### Study cohorts

The current meta-analysis included a total of 44 871 women of European ancestry from two cohorts. We used the data of the UKBB, a population-based biobank comprising 502 637 people (aged 37–73 years) recruited from across the UK during 2006–2010, who have filled out detailed medical history questionnaires (41). Menstrual cycle length information was derived from data field 3710 ‘Length of menstrual cycle’. Participants were asked ‘How many days is your usual menstrual cycle? (The number of days between each menstrual period)’. This question was asked of women who had indicated they were not menopausal and still had menstrual periods in their answer to data field 2724 [‘Have you had your menopause (periods stopped)?’]. The phenotype was transformed according to the default PHESANT pipeline (42), whereby the integer phenotype is split into three ordered bins if a single value represents >20% of all respondents answers. As a result, length of menstrual cycle was split into <26, 26–28 and ≥28 days. All answers corresponding to ‘Irregular cycle’, ‘Do not know’ and ‘Prefer not to answer’ were coded as NA. As a result, each bin included 14 211 (mean age, 45.7 years; range, 39–69 years), 4949 [45.7 (40–70) years], and 29227 [45.9 (40–70) years] individuals, respectively. Additionally, individuals were filtered as described in https://github.com/Nealelab/UK_Biobank_GWAS, leaving 30 245 individuals for final analysis.

We also included data from the Estonian Biobank (EGCUT), a population-based biobank with 51 515 participants of European ancestry (43). In EGCUT, women >35 years were asked about their menstrual cycle length using the question ‘Approximately how long was your menstrual cycle when you were between 25 and 35 years old?’, with the following choices: ‘I don’t know‘, ‘I have not had any menstrual cycles’, ‘Irregular’, ‘20 days or less’, ‘21–24 days’, ‘25–29 days’, ‘30–135 days’ or ‘more than 35 days’. To follow a similar structure as with the UKBB data, the answers were regrouped into three bins: <25, 25–29 and ≥30 days, resulting in 2877 [56.3 (33–95) years], 10 354 [54.3 (33–101) years] and 1395 [50.9 (34–96) years] individuals in each bin, respectively.

### GWAS and meta-analysis

In the UKBB data set, quality control and association testing were carried out as described in https://github.com/Nealelab/UK_Biobank_GWAS. In brief, samples were filtered for white British genetic ancestry, related individuals, individuals with sex chromosome aneuploidies and individuals who had withdrawn their participation in the UKBB. The analysis included SNPs imputed to the Haplotype Reference Consortium (HRC) reference panel, and additional filters included minor allele frequency (MAF) > 0.1%, Hardy–Weinberg equilibrium (HWE) *P* > 1 $\times$ 10^-10^ and imputation INFO score > 0.8. Association testing was carried out using linear regression implemented in HAIL (https://github.com/hail-is/hail), adjusting for the first 10 principal components (PCs).

In EGCUT, Illumina Human CoreExome, OmniExpress, 370CNV BeadChip and GSA arrays were used for genotyping. Quality control included filtering on the basis of sample call rate (<98%), heterozygosity (> mean ± 3SD), genotype and phenotype sex discordance, cryptic relatedness (IBD > 20%) and outliers from the European descent based on the MDS plot in comparison with HapMap reference samples. SNP quality filtering included call rate (<99%), MAF (<1%) and extreme deviation from HWE (*P* < 1 $\times$ 10^−4^). Imputation was performed using SHAPEIT2 for prephasing, the Estonian-specific reference panel [PMID: 28401899] and IMPUTE2 [PMID: 19543373] with default parameters. Association testing was carried out with EPACTS (https://github.com/statgen/EPACTS), adjusting for 10 PCs and age at recruitment.

Before meta-analysis, results from individual cohorts underwent central quality control with EasyQC (44), checking for allele frequency against the HRC reference and filtering out variants with a MAF < 1% and INFO score < 0.4. The results from individual cohorts were meta-analyzed with METAL (45) using sample-size weighted *P*-value-based meta-analysis with genomic control correction. The meta-analysis included 9 344 826 markers, and those with a *P* < 5 $\times$ 10^-8^ were considered genome-wide significant.

To convert the effects obtained from the linear regression of binned trait to a standardized scale, we calculated the mean and variance of the 0, 1 and 2 binned menstrual cycle length phenotype and divided the effect estimates from linear regression with calculated standard deviation of the binned phenotype.

### Gene-based testing

Gene-based genome-wide association analysis was carried out with MAGMA 1.6 (14) with default settings implemented in FUMA (21). Briefly, variants were assigned to protein-coding genes (*n* = 18 297; Ensembl build 85) if they are located in the gene body, and the resulting SNP *P*-values are combined into a gene test-statistic using the SNP-wise mean model (14). According to the number of tested genes, the level of genome-wide significance was set at 0.05/18 297 = 2.7 $\times$ 10^-6^.

### Heritability estimate

The menstrual cycle length GWAS meta-analysis summary statistics and LDSC method (13) were used for heritability estimation. The LD estimates from European ancestry samples in the 1000 Genomes Project were used as a reference.

### Functional mapping

Functional annotation was performed using the FUMA platform designed for prioritization, annotation and interpretation of GWAS results (21). As the first step, independent significant SNPs in the GWAS meta-analysis summary statistics were identified based on their *P*-values (*P* < 5 $\times$ 10^-8^) and independence from each other (*r*^2^ < 0.6 in the 1000G phase 3 reference) within a 1Mb window. Thereafter, lead SNPs were identified from independent significant SNPs, which are independent of each other (*r*^2^ < 0.1). SNPs that were in LD with the identified independent SNPs (*r*^2^$\ge$ 0.6) within a 1Mb window, have a MAF of $\ge$ 1% and GWAS meta-analysis *P*-value of >0.05 were selected as candidate SNPs and taken forward for further annotation.

FUMA annotates candidate SNPs in genomic risk loci based on functional consequences on genes Annotate Variation (ANNOVAR) (46), CADD (a continuous score showing how deleterious the SNP is to protein structure/function; scores >12.37 indicate potential pathogenicity) (47) and RegulomeDB scores (ranging from 1 to 7, where lower score indicates greater evidence for having regulatory function) (48), 15 chromatin states from the Roadmap Epigenomics Project (49,50), eQTL data (GTEx v6 and v7) (22), blood eQTL browser (51), BIOS QTL browser (52), BRAINEAC (53), MuTHER (54), xQTLServer (55) and the CommonMind Consortium (23) and 3D chromatin interactions from HI-C experiments of 21 tissues/cell types (56), also embedded in the FUMA platform. Next, genes were mapped using positional mapping, which is based on ANNOVAR annotations and maximum distance between SNPs (default 10 kb) and genes, eQTL mapping and chromatin interaction mapping. Chromatin interaction mapping was performed with significant chromatin interactions (defined as FDR < 1 $\times$ 10^-6^). The two ends of significant chromatin interactions were defined as follows: region 1, a region overlapping with one of the candidate SNPs; and region 2, another end of the significant interaction, used to map to genes based on overlap with a promoter region (250 bp upstream and 50 bp downstream of the transcription start site).

### Genetic associations between menstrual cycle length and other traits

The Oxford BIG Server (v2.0; http://big.stats.ox.ac.uk/) was used to query the sentinel variants in each locus against an array of UKBB phenotypes (Supplementary Material, Table 3). Additionally, during the FUMA functional mapping, sentinel SNPs and proximal SNPs in tight LD (*r^2^* = 0.6) were linked with the GWAS catalog (https://www.ebi.ac.uk/gwas/). Full results of the GWAS catalog query are shown in Supplementary Material, Table 2.

We analyzed genome-wide genetic correlation analyses applying the LDSC method (13) using the LD-Hub resource and 50 selected traits (cardiometabolic, anthropometric, autoimmune, hormone, reproductive, cancer and aging categories). Full results of the LDSC genetic correlation analysis are reported in Supplementary Material, Table 5.

### Tissue specificity and gene set enrichment analyses

Tissue and gene set enrichment analyses were carried out with GENE2FUNC implemented in FUMA (21). Genes that were highlighted in MAGMA gene-based analysis or which had functional annotation support from eQTL and chromatin interaction data were used as an input (a total of 14 genes). Using all genes as a background gene set, 2 $\times$ 2 enrichment tests were carried out. The GTEx v7 30 general tissue types data set was used for tissue specificity analyses. DEG sets are pre-calculated in the GENE2FUNC by performing two-sided *t*-test for any one of tissues against all others. For this, expression values were normalized (zero-mean) following a log_2_ transformation of expression values (transcripts per million). Genes with *P* ≤ 0.05 after Bonferroni correction and absolute log fold change ≥0.58 were defined as DEGs in a given tissue compared to others. In addition to general DEG, upregulated and downregulated DEG sets were also pre-calculated by taking sign of *t*-statistics into account. Our set of prioritized input genes was tested against each of the DEG sets using a hypergeometric test, where background genes are genes that have average expression value > 1 in at least one of the tissues. Significant enrichment at Bonferroni corrected *P* ≤ 0.05 are colored in red in Supplementary Material, Figure 4.

#### Tools used in this paper

HAIL: https://github.com/hail-is/hail

Oxford BIG browser: http://big.stats.ox.ac.uk/

FUMA: http://fuma.ctglab.nl/

GWAS catalog: https://www.ebi.ac.uk/gwas/

GREAT: http://great.stanford.edu/public/html/

HaploReg: http://archive.broadinstitute.org/mammals/haploreg/haploreg.php

MGI: http://www.informatics.jax.org/phenotypes.shtml

## Supplementary Material

Supplementary Data
