# Supplementary material for: Large-scale meta-analysis highlights the hypothalamic–pituitary–gonadal axis in the genetic regulation of menstrual cycle length
Source: Hum Mol Genet. 2018 Sep 7;27(24):4323–32. doi: 10.1093/hmg/ddy317 (PMC6276838; doi:10.1093/hmg/ddy317)
Supplement: Supplementary Data [file ddy317_supp.zip › Supplementary_Figures.pdf]

Supplementary Figures

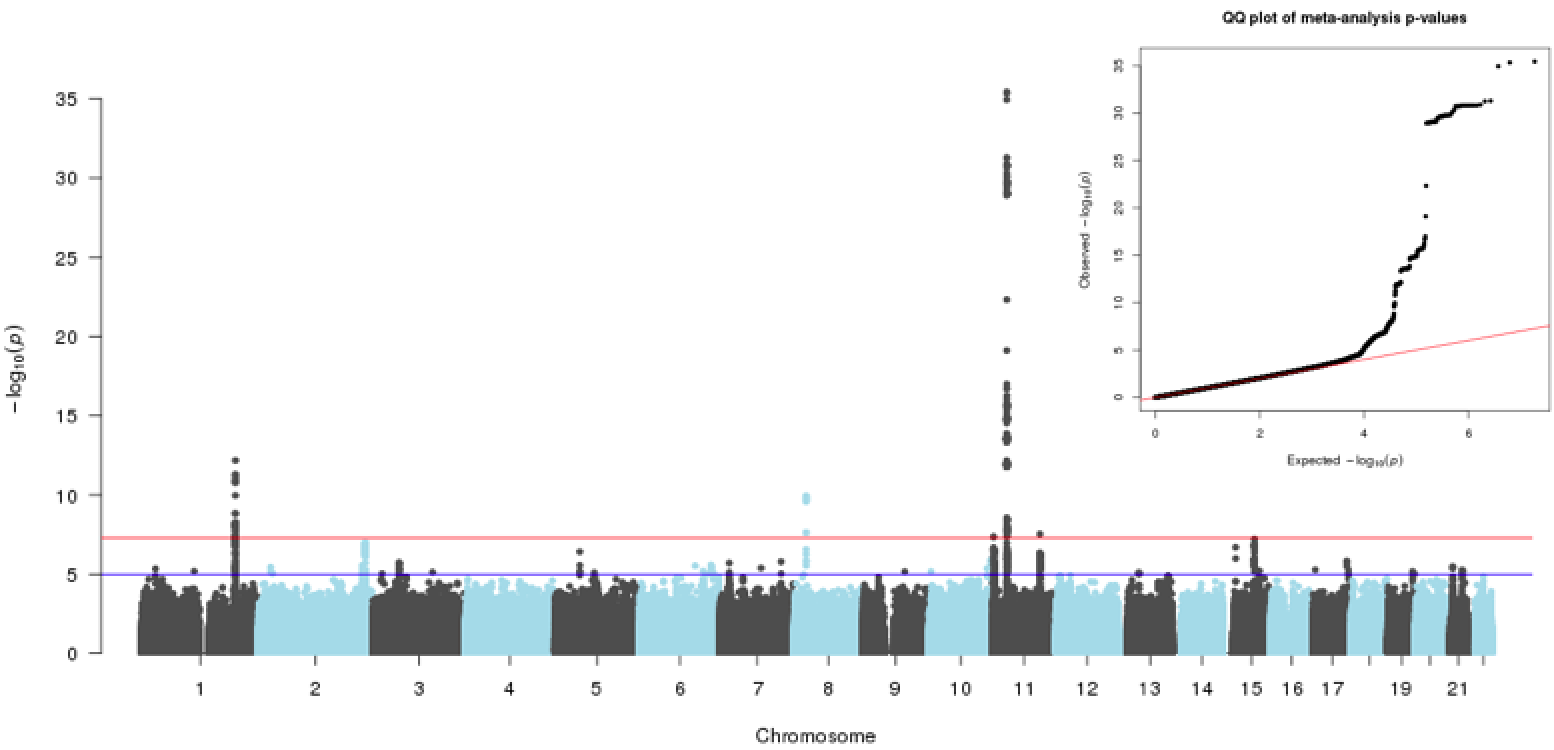

Supplementary Figure 1. Manhattan and QQ plot for menstrual cycle length GWAS meta-analysis.

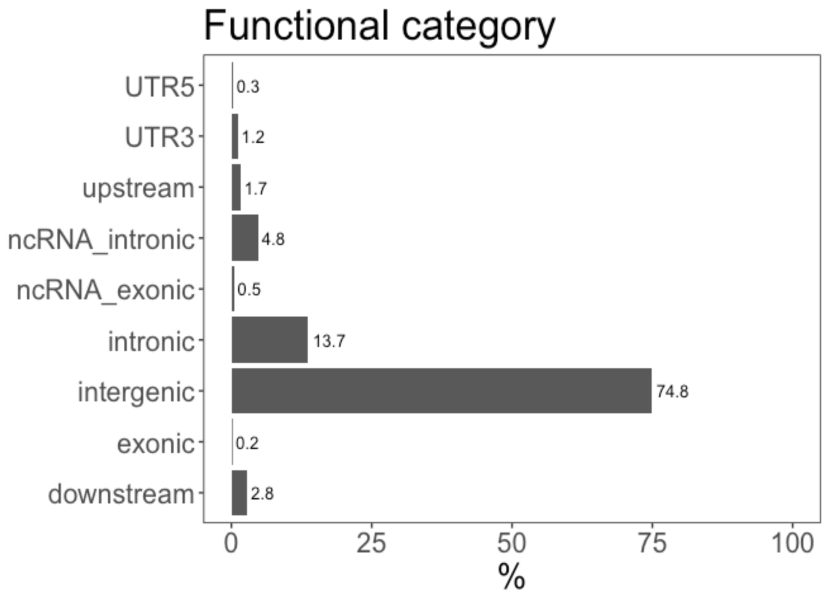
Functional category
UTR5
UTR3
upstream
ncRNA_intronic
ncRNA_exonic
intronic
intergenic
exonic
downstream
0.3
1.2
1.7
4.8
0.5
13.7
74.8
0.2
2.8
0
25
50
75
100
%

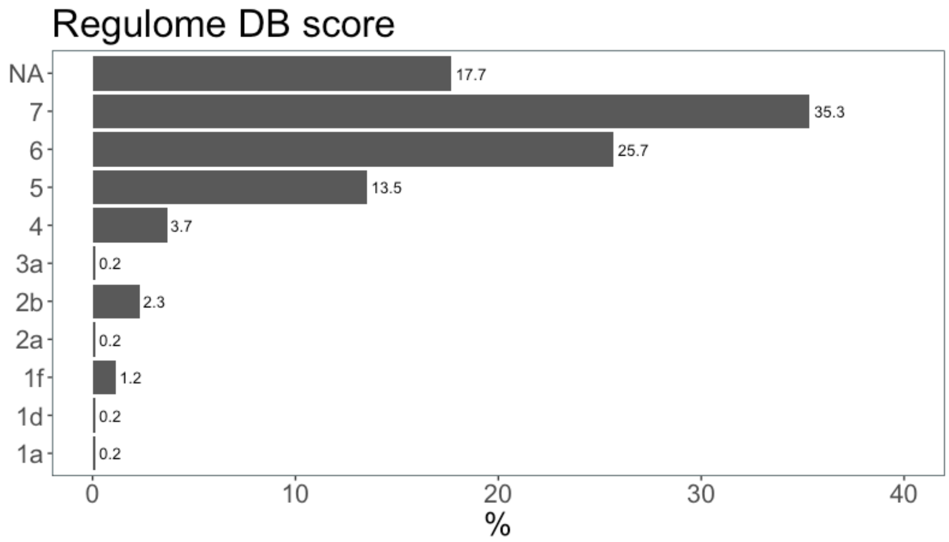
Regulome DB score
NA
7
6
5
4
3a
2b
2a
1f
1d
1a
17.7
35.3
25.7
13.5
3.7
0.2
2.3
0.2
1.2
0.2
0.2
0
10
20
30
40
%

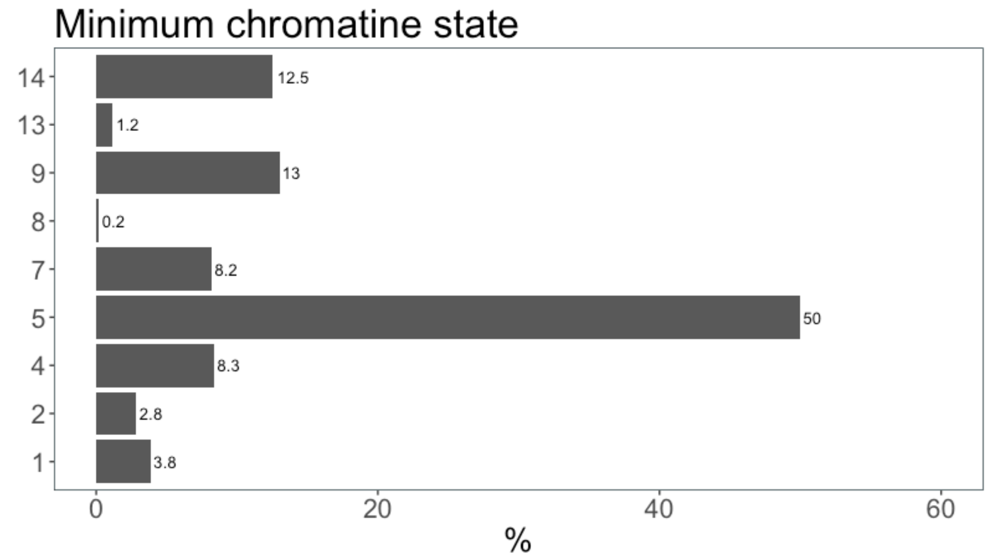

Supplementary Figure 2. Functional annotation for all SNPs with $r^2 \geq 0.6$ with the sentinel SNPs. a) Percentage of SNPs according to their functional category; b) Percentage of SNPs according to their RegulomeDB score. Lower score indicates a more likely regulatory role; c) Percentage of SNPs according to their minimum chromatin state across 127 tissues. Lower score indicates a more likely regulatory role.

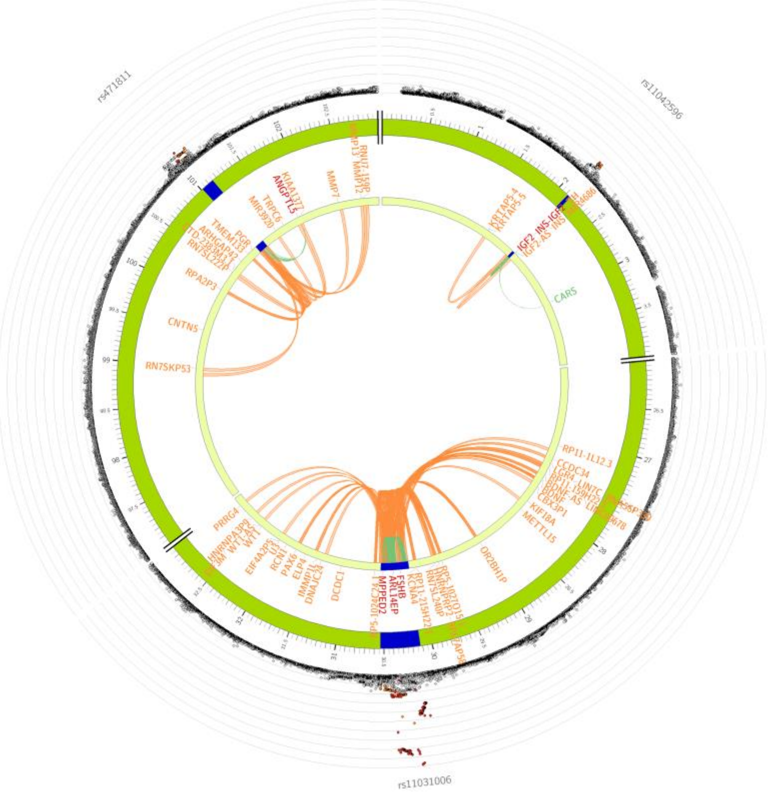
rs471811
rs11042596
rs11031006
MMP7
ANGPTL5
TRPC6
RPA2P3
CNTN5
RN7SKP53
PRRG4
DCDC1
CARS
RP11-1L12.3
CCDC34
KIF18A
METTL15
OR2BH1P

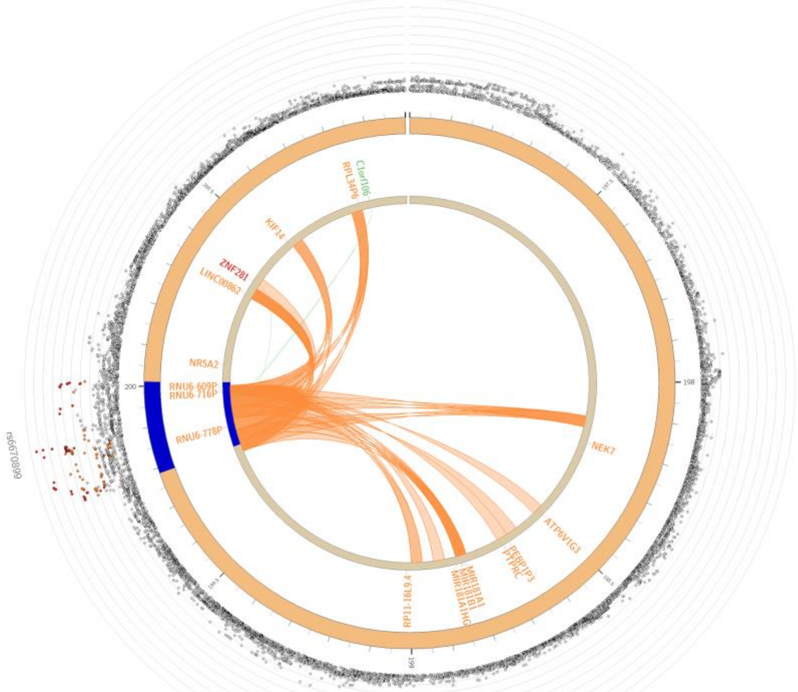
rs6670899
C1orf106
RPL34P6
KIF14
ZNF281
LINC00862
NR5A2
RNU6-609P
RNU6-716P
RNU6-778P
NEK7
ATP6V1G3

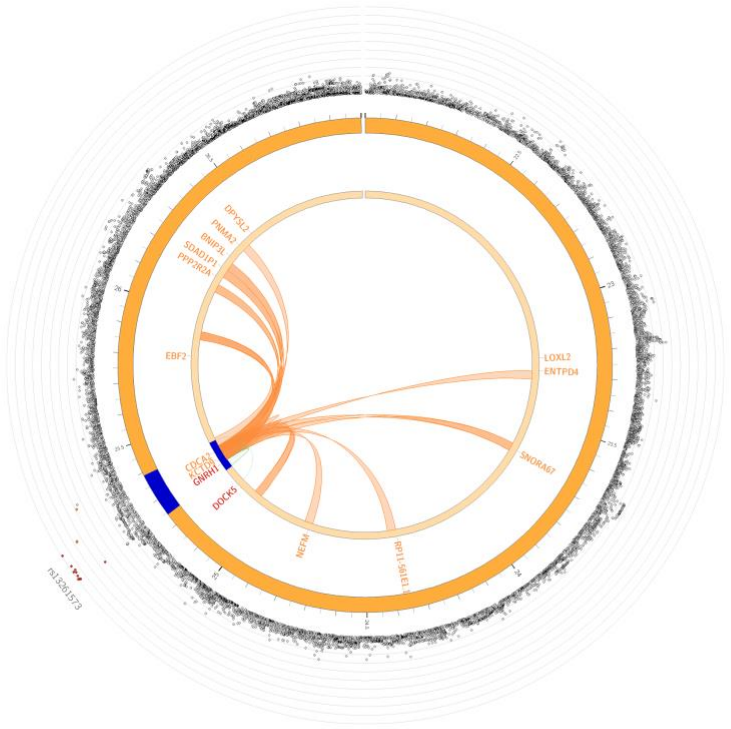

Supplementary Figure 3. Circos plots demonstrating the results of eQTL and chromatin interaction mapping for loci on chromosome 11, 1, and 8, respectively. The outermost layer depicts SNPs with a $P < 0.05$ in a Manhattan-style plot. SNPs in detected genomic risk locus are coloured according to their r^2 value to the most significant variant at the locus. Y-axis values range from 0 to the maximum –log10(P-value) of the SNPs. The second and third layer represent the chromosome ring, where the risk locus is coloured in blue, and genes mapped by chromatin interactions with this region, depicted by orange ribbons, are displayed. eQTL associations are shown by green ribbons, and genes that were mapped by both eQTL associations and chromatin interaction data are highlighted in red.

A)

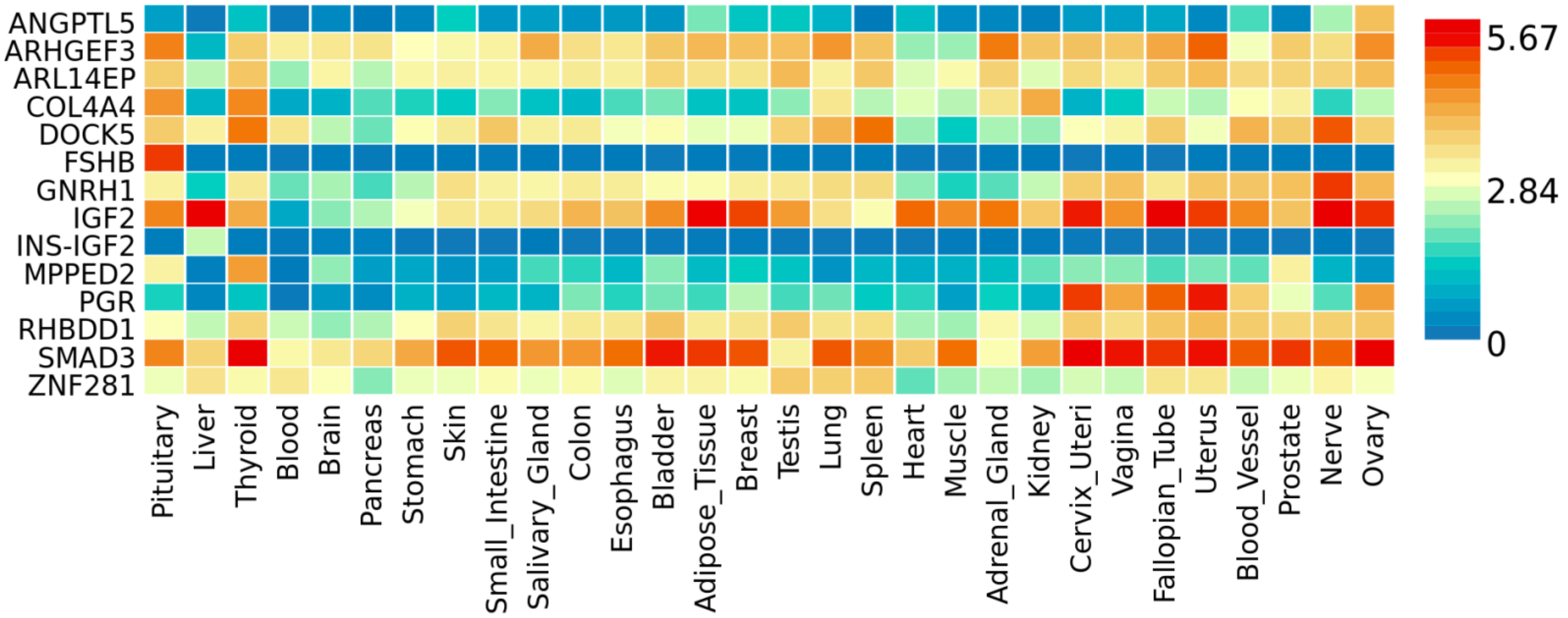
ANGPTL5
ARHGEF3
ARL14EP
COL4A4
DOCK5
FSHB
GNRH1
IGF2
INS-IGF2
MPPED2
PGR
RHBDD1
SMAD3
ZNF281
Pituitary
Liver
Thyroid
Blood
Brain
Pancreas
Stomach
Skin
Small_Intestine
Salivary_Gland
Colon
Esophagus
Bladder
Adipose_Tissue
Breast
Testis
Lung
Spleen
Heart
Muscle
Adrenal_Gland
Kidney
Cervix_Uteri
Vagina
Fallopian_Tube
Uterus
Blood_Vessel
Prostate
Nerve
Ovary
5.67
2.84
0

B)

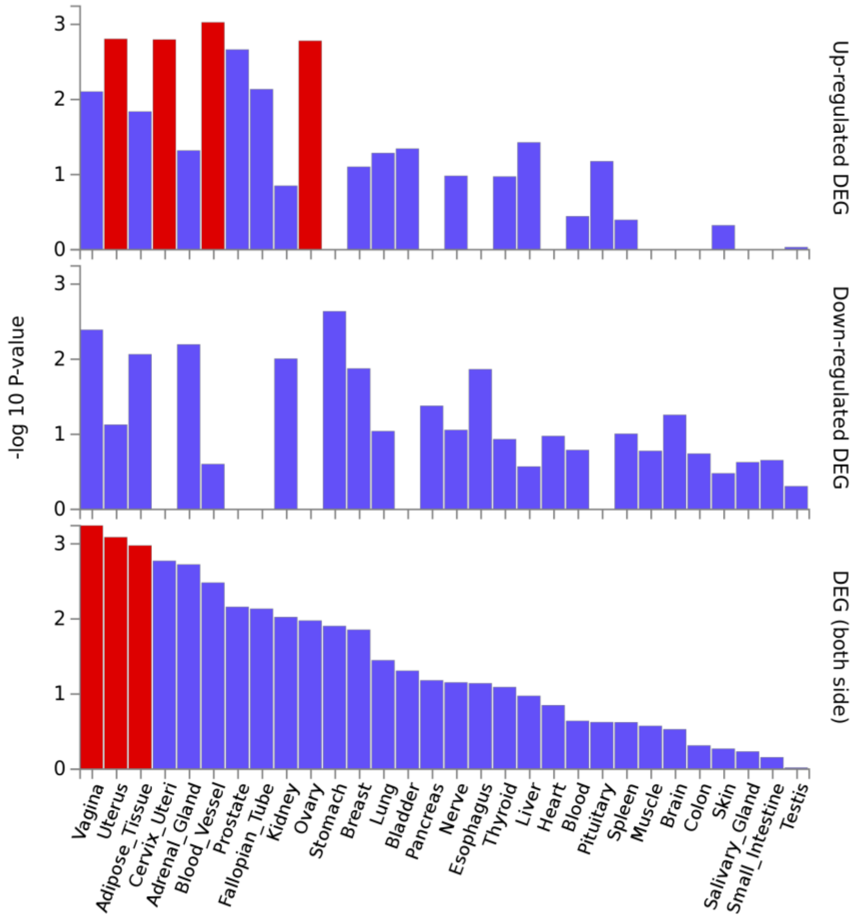

Supplementary Figure 4. a) Heatmap displaying average expression of prioritized genes and b) differential expression analysis of prioritized genes across GTEx v7 30 general tissue types.
